# Identification of Rapeseed MicroRNAs Involved in Early Stage Seed Germination under Salt and Drought Stresses

**DOI:** 10.3389/fpls.2016.00658

**Published:** 2016-05-13

**Authors:** Hongju Jian, Jia Wang, Tengyue Wang, Lijuan Wei, Jiana Li, Liezhao Liu

**Affiliations:** Chongqing Engineering Research Center for Rapeseed, College of Agronomy and Biotechnology, Southwest UniversityChongqing, China

**Keywords:** drought stress, salt stress, microRNA, *Brassica napus*, seed germination

## Abstract

Drought and salinity are severe and wide-ranging abiotic stresses that substantially affect crop germination, development and productivity, and seed germination is the first critical step in plant growth and development. To comprehensively investigate small-RNA targets and improve our understanding of miRNA-mediated post-transcriptional regulation networks during *Brassica napus* seed imbibition under drought and salt stresses, we constructed three small-RNA libraries from *B. napus* variety ZS11 embryos exposed to salt (200 mM NaCl, denoted “S”), drought (200 g L^−1^ PEG-6000, denoted “D”), and distilled water (denoted “CK”) during imbibition and sequenced them using an Illumina Genome Analyzer. A total of 11,528,557, 12,080,081, and 12,315,608 raw reads were obtained from the CK, D, and S libraries, respectively. Further analysis identified 85 known miRNAs belonging to 31 miRNA families and 882 novel miRNAs among the three libraries. Comparison of the D and CK libraries revealed significant down-regulation of six miRNA families, miR156, miR169, miR860, miR399, miR171, and miR395, whereas only miR172 was significantly up-regulated. In contrast, comparison of the S library with the CK library showed significant down-regulation of only two miRNA families: miRNA393 and miRNA399. Putative targets for 336, 376, and 340 novel miRNAs were successfully predicted in the CK, D, and S libraries, respectively, and 271 miRNA families and 20 target gene families [including disease resistance protein (DIRP), drought-responsive family protein (DRRP), early responsive to dehydration stress protein (ERD), stress-responsive alpha-beta barrel domain protein (SRAP), and salt tolerance homolog2 (STH2)] were confirmed as being core miRNAs and genes involved in the seed imbibition response to salt and drought stresses. The sequencing results were partially validated by quantitative RT-PCR for both conserved and novel miRNAs as well as the predicted target genes. Our data suggest that diverse and complex miRNAs are involved in seed imbibition, indicating that miRNAs are involved in plant hormone regulation, and may play important roles during seed germination under salt- or drought-stress conditions.

## Introduction

Drought and salinity are severe and wide-ranging abiotic stresses that substantially affect the germination, development, and productivity of crops. Unlike most animals, plants are sessile organisms that have evolved mechanisms to cope with a wide range of changes in the environment and climate through the regulation of gene expression (Sunkar, 2010). These mechanisms involve complicated biological processes that finely coordinate molecular signaling pathways. To prevent cellular damage and to aid in recovery, specific genes, or proteins are activated at the molecular level by various stresses (Chinnusamy et al., 2007; Shinozaki and Yamaguchi-Shinozaki, 2007). Although there are specific pathways for each stress, certain key genes, or proteins act as nodal components that are integral in stress responsive signal transduction pathways (Knight and Knight, 2001; Fujita et al., 2006). Much effort has been devoted to elucidating gene expression in plants exposed to dry and brackish conditions; however, the mechanisms underlying the regulation of gene expression under these conditions remain largely unknown.

Various types of short and long non-coding RNAs (ncRNAs) are of great interest to molecular biologists. Among ncRNA subclasses, microRNAs (miRNAs), which are endogenous, non-coding RNAs ~21 nucleotides in length that negatively regulate gene expression at the transcriptional, post-transcriptional, and translational levels in both plants and animals (Bartel, 2004; Brodersen et al., 2008; Yang X. Y. et al., 2013), are the most thoroughly characterized (Bartel, 2009). The first discovered miRNAs were *lin-4* and *let-7*, which control developmental timing in *Caenorhabditis elegans* and were later identified in almost all multicellular eukaryotes (Lee et al., 1993). In plants, miRNAs are involved in controlling many biological and metabolic processes, including organ maturation (Juarez et al., 2004; Guo et al., 2005), hormone signaling (Liu et al., 2009), developmental timing (Achard et al., 2004), and responses to pathogens (Sullivan and Ganem, 2005; Navarro et al., 2006) as well as to environmental abiotic stresses such as drought (Zhao et al., 2007), salinity (Zhao et al., 2009), heavy metals (Huang et al., 2009), and cold (Zhou et al., 2008). For example, miR159 and miR160 have been shown to be involved in the regulation of seed germination through effects on the sensitivity of seeds to abscisic acid (ABA) and auxin, suggesting that these miRNAs may function in the seed germination process (Reyes and Chua, 2007; Nonogaki, 2010). Many other miRNAs have also been shown to act under conditions of environmental stress, such as miR169 with high salt (Zhao et al., 2009), miR395 with sulfate starvation (Jones-Rhoades and Bartel, 2004), and miR398 with heavy metal toxicity (Sunkar et al., 2006). To date, more than 40 miRNA families have been observed to be involved in responses to abiotic stresses in plants (Sunkar, 2010), many of which are involved in responses to salt and drought stresses (Xiong et al., 2006; Peng et al., 2014). In addition, some miRNAs have been identified in more than one plant species (Sunkar et al., 2007), suggesting that their function in response to stress might be conserved among species, whereas others, called non-conserved or novel microRNAs, are species or tissue specific.

*Brassica napus* L. (AACC, 2*n* = 38), commonly known as rapeseed, is an amphidiploid species that originated from interspecies crosses between *Brassica rapa* (AA, 2*n* = 20) and *Brassica oleracea* (CC, 2*n* = 18). *B. napus* is the third most economically important member of the genus *Brassica*, provides many useful products, and is grown worldwide (Dalton-Morgan et al., 2014). Similar to all crops, exposure to soil salinity and drought stresses at specific developmental stages in *Brassica* leads to compromised growth, development, and seed production. Although miRNAs and their targets have been widely studied in model plants, there is only limited knowledge to date on the small-RNA population of rapeseed. In the first report of miRNAs in *Brassica*, Xie et al. (2007) identified 21 miRNAs in *B. napus* using computational methods (Xie et al., 2007). Pant et al. (2009) performed the first deep sequencing of small-RNA libraries to identify phosphate deprivation-responsive miRNAs in *B. rapa* and *Arabidopsis* (Pant et al., 2009). A study of the global miRNA response to phosphate deficiency and cadmium stress in *B. napus* was conducted by Huang et al. (2010), validating *BnAPS3, BnAPS4*, and *BnSultr2:1* as targets of miR395. Subsequently, both conserved and *Brassica*-specific miRNAs were reported in studies of *B. rapa* (Kim et al., 2012; Wang F. D. et al., 2012; Yu et al., 2012), *B. napus* (Korbes et al., 2012; Wang J. Y. et al., 2012; Xu et al., 2012; Zhao et al., 2012; Zhou et al., 2012), *B. oleracea* (Wang J. Y. et al., 2012), and *Brassica juncea* (Yang J. H. et al., 2013).

Seed germination is the first critical step in plant growth and development. Plant development is determined by a multitude of factors that include genetic makeup and both biological and non-biological challenges. As abiotic stresses, such as drought, salt, heat, cold, and heavy metals, are major factors affecting seed germination, it is important to study the regulatory molecules and associated gene networks of seed germination under drought and salt stresses to improve crop yields via biotechnology, particularly with respect to the possible small RNA-mediated regulation of seed germination under such stress (Martin et al., 2010). miRNA expression during seed germination has been characterized in maize (Wang et al., 2011) and *Arabidopsis thaliana* (Jung and Kang, 2007; Kim et al., 2010) by analyzing conserved miRNAs derived from miRBase (Griffiths-Jones et al., 2008) through high-throughput sequencing. Since the initial report on rapeseed miRNAs, many conserved and rapeseed-specific miRNAs in different tissues have been identified as being involved in different biological processes (Buhtz et al., 2008; Huang et al., 2010; Korbes et al., 2012; Xu et al., 2012; Zhao et al., 2012; Zhou et al., 2012). However, the roles of miRNAs during *B. napus* seed germination under drought and salt stresses remain largely unknown. Potential information about miRNA mediated regulation under salt and drought stresses in rapeseed which may help to explore genomics behind stress mediated response.

To investigate small-RNA targets comprehensively and to further understand the miRNA-mediated post-transcriptional regulation network during rapeseed germination under drought and salt stresses, we constructed three small-RNA libraries from embryos of *B. napus* variety ZS11 seeds undergoing 60 h of imbibition under salt (S) or drought (D) stress compared to distilled water (CK) and then performed miRNA sequencing. The miRNAs and targets identified in this study may facilitate an understanding of the molecular mechanisms of stress signaling.

## Materials and methods

### Plant material, RNA isolation, and small-RNA high-throughput sequencing

Healthy *B. napus* ZS11 seeds were surface sterilized and soaked using filter paper as the underlay in distilled water, 200 mM NaCl, or 200 g L^−1^ PEG-6000 solution, marked CK, S, and D, respectively; three replicates were performed. The seeds were incubated at 22°C in darkness for 60 h until the hypocotyl emerged but the length was < 2 mm. The embryo and hypocotyl together were frozen in liquid nitrogen immediately after collection and then stored at −80°C. Total RNA was isolated using TRIzol H (Invitrogen, USA) according to the manufacturer's instructions. The quality of the RNA samples was evaluated as described previously (Han et al., 2014). Three RNA samples were sent to BGI (Shenzhen, China) for sRNA library construction and Solexa sequencing using the Illumina HiSeq 2000 platform according to the manufacturer's protocols (Liang et al., 2010).

### Small-RNA data analysis

Small-RNA reads of each sample were filtered with the SOAPnuke software (http://soap.genomics.org.cn/) (Li et al., 2009), developed by the Beijing Genome Institute (BGI), to remove low-quality reads, reads smaller than 18 nt, adaptor sequences, and contamination formed by adaptor–adaptor ligation according to the software's default settings (http://soap.genomics.org.cn). The Rfam database (http://www.sanger.ac.uk/software/Rfam) (Griffiths-Jones et al., 2005) and the GenBank noncoding RNA database (http://www.ncbi.nlm.nih.gov/) were used to annotate these perfectly matched sequences, which might include noncoding RNAs (e.g., tRNA, rRNA, snRNA, and snoRNA) or mRNA degradation fragments. Subsequently, small RNAs were aligned to miRNA precursors of rapeseed in miRBase 21.0 (Kozomara and Griffiths-Jones, 2014) to obtain known miRNAs with the following two criteria: (1) the small RNA tags align to the miRNA precursors in miRBase with no mismatches; (2) after fulfilling the first criterion, the tags align to mature miRNAs in miRBase with at least 16 nt of overlap, allowing for offsets. Those miRNAs that satisfied both the above criteria were counted to determine the expression of known miRNAs. In addition, sequencing data of three samples were uploaded to NIH Short Read Archive with accession number SRP073343.

### Prediction of novel miRNAs

To identify novel miRNAs in rapeseed, small-RNA sequences were aligned to the *B. napus* genome sequence to obtain precursor sequences. Novel miRNA candidates were identified with the Mireap program developed by the BGI. The sequences were regarded as novel miRNA candidates only if their structures met the previously reported criteria (Allen et al., 2005; Wang et al., 2011). Briefly, the secondary structures of filtered pre-miRNA sequences were checked using Mfold (Wiese and Hendriks, 2006). In each case, only the structure exhibiting the minimum free energy was selected for manual inspection.

### Target gene prediction

The psRobot software (Wu et al., 2012) was used to predict the potential targets of newly identified miRNAs responsive to salt and drought stresses. All predicted target genes were assessed using the scoring system and according to previously described criteria (Srivastava et al., 2014). Genes with a score < 2.5 were considered miRNA targets.

### Expression analysis of rapeseed miRNAs using qRT-PCR

Samples of embryos were collected from the CK, D, and S treatments via the same method used for miRNA library construction. Total RNA from each sample was extracted as described above. miRNA abundance was checked by qPCR according to previous reports (Shi and Chiang, 2005). The addition of poly(A) tails to sRNAs by poly(A) polymerase and cDNA synthesis was carried out using miRcute miRNA First-Strand cDNA Synthesis Kit (TIANGEN, Beijing, China), and qPCR was performed with miRcute miRNA qPCR Detection Kit according to the manufacturer's instructions. qPCR was performed using a CFX96 Real-time System (BIO-RAD, USA) with SYBR® Premix (TIANGEN, Beijing, China). The miRNA-specific forward primer for each miRNA was designed based on the entire miRNA sequence with the Tm adjusted to 60°C (Table S1). The universal reverse primer was designed based on the adapter sequence, which was provided with the miRNA cDNA synthesis kit (TIANGEN, Beijing, China). The *B. napus* U6 gene was used as the internal control to normalize the qPCR data, as previously described (Huang et al., 2010). The PCR conditions were as follows: 95°C for 2 min, followed by 40 cycles of 95°C for 20 s and 60°C for 30 s. A final ramping stage of 65–95°C was performed to confirm the absence of multiple products and primer dimers. The fold-changes in miRNA expression level were calculated using the comparative Ct (2^−ΔΔCt^) method and normalized against the expression level in the CK sample. Triplicate biological and technological replicates were performed for the three samples.

### Validation of predicted miRNA target gene expression profiles by quantitative RT-PCR

Predicted target genes were obtained from the *B. napus* Sequence database (http://www.genoscope.cns.fr/brassicanapus/). The software Primer Premier 5.0 (PREMIER Biosoft Int., Palo Alto, CA, USA) was used to design specific primers for quantitative RT-PCR (Table S2), and qPCR was performed using a CFX96 Real-time System (BIO-RAD, USA) with SYBR® Premix (TIANGEN, Beijing, China). Briefly, each 20 μL PCR reaction contained ~100 ng cDNA, 8 μL 2.5 × RealMasterMix/20 × SYBR solution, and 200 nM each primer. The PCR conditions were as follows: 94°C for 2 min, followed by 40 cycles of 94°C for 15 s and 60 °C for 30 s. A final ramping stage of 65–95°C was performed to confirm the absence of multiple products and primer dimers. Actin was used for each sample as an endogenous control. All samples were subjected to at least three technical replicates. Data were analyzed using the Ct (2^−ΔΔCt^) method described above. Moreover, the software SPSS19.0 (SPSS Inc., Chicago, IL, USA) was used for statistical analysis in this study.

## Results

### Sequence analysis of short RNAs

To identify and characterize conserved and novel miRNAs involved in *B. napus* seed germination in response to salt and drought stresses, we constructed three small-RNA libraries and obtained sequence reads by Illumina high-throughput sequencing. A total of 11,528,557, 12,080,081, and 12,315,608 raw reads were obtained from the control, drought, and salt stress treatments, respectively. After the removal of low-quality reads, 3′ adapters and 5′ adapters, reads shorter than 18 nt, and other contaminating sequences, 11,317,915 (98.68%), 11,917,020 (99.17%), and 12,087,933 (98.66%) clean reads representing 3,754,707, 4,246,335, and 3,658,598 unique sequences were obtained, respectively (Table S3). Additionally, we annotated all 18–28 nt reads from the CK, D, and S libraries and obtained 4,534,861, 5,095,273, and 4,454,112 unique reads, respectively (Table 1). Approximately 2.74, 1.59, and 2.24% of the unique reads matched noncoding RNAs (rRNAs, tRNAs, snRNAs, snoRNAs), accounting for 11.06, 10.84, and 16.05% of the total reads in the CK, D, and S libraries, respectively (Table 1). Subsequently, ~15.38, 14.99, and 15.54% of the reads in CK, D, and S libraries, respectively, were mapped to coding sequences, which may constitute RNA degradation products, whereas a very small percentage of reads were mapped to intron sequences, which may be able to produce pre-miRNAs.

**Table 1:** **Distribution of small RNAs among different categories in three libraries**.

**Locus class**	**CK**				**D**				**S**			
	**Total**		**Unique**		**Total**		**Unique**		**Total**		**Unique**	
**NONPROTEIN-CODING RNAs**												
snoRNA	5352	0.05%	2239	0.05%	6645	0.06%	2802	0.05%	5057	0.04%	2252	0.05%
snRNA	8346	0.07%	3384	0.07%	9371	0.08%	3646	0.07%	9674	0.08%	3586	0.08%
tRNA	216,253	1.91%	7683	0.17%	167,375	1.40%	7226	0.14%	267,363	2.21%	8886	0.20%
rRNA	1,021,790	9.03%	65,548	1.45%	1,108,552	9.30%	67,536	1.33%	1,657,908	13.72%	84,936	1.91%
**SMALL RNAs MATCHING PROTEIN-CODING GENES**												
exon_antisense	242,071	2.14%	106,937	2.36%	278,336	2.34%	120,562	2.37%	248,569	2.06%	108,882	2.44%
exon_sense	533,661	4.72%	148,056	3.26%	525,008	4.41%	160,432	3.15%	567,580	4.70%	159,510	3.58%
intron_antisense	368,034	3.25%	175,881	3.88%	430,309	3.61%	195,244	3.83%	366,290	3.03%	169,262	3.80%
intron_sense	722,449	6.38%	266,756	5.88%	809,310	6.79%	287,415	5.64%	705,803	5.84%	254,553	5.72%
miRNA	2,131,604	18.83%	503	0.01%	1,556,356	13.06%	511	0.01%	2,123,566	17.57%	496	0.01%
repeat	5537	0.05%	3060	0.07%	6978	0.06%	3521	0.07%	6125	0.05%	3114	0.07%
unannotation	6,062,818	53.57%	3,754,707	82.80%	7,018,780	58.90%	4,246,335	83.34%	6,129,998	50.71%	3,658,598	82.14%
Total	11,317,915	100%	4,534,754	100%	11,917,020	100%	5,095,230	100%	12,087,933	100%	4,454,075	100%

*CK, control; D, drought stress; S, salt stress*.

**Figure 1:**
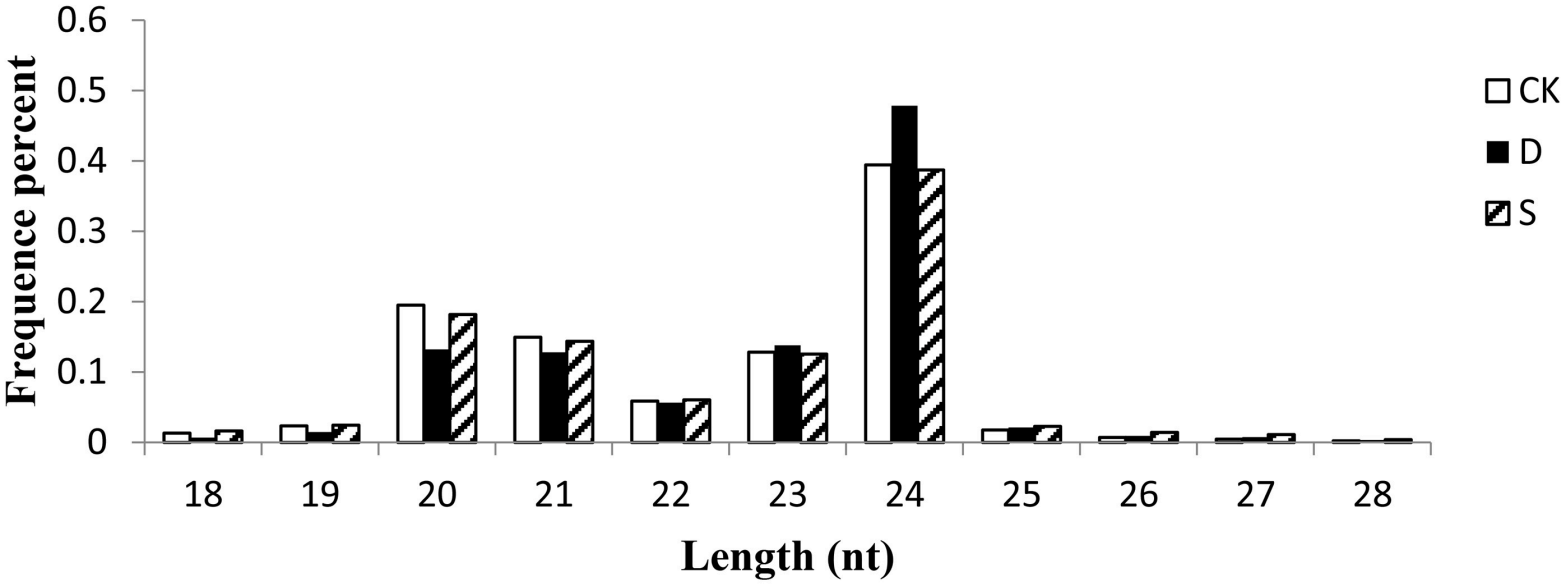
**Size distribution of small RNAs in CK, D, and S libraries from rapeseed**. CK, control; D, drought stress; S, salt stress.

The distribution of various sizes of the small RNAs was not homogeneous. The most abundant were small RNAs 20–24 nt in length (Figure 1), among which sequences 24 nt long were in the greatest abundance, accounting for 39.42, 47.82, and 38.69% of the sequences in the control, drought, and salt libraries, respectively. These results are similar to those in other plant species, including *A. thaliana* (Rajagopalan et al., 2006)*, Arachis hypogaea* (Zhao et al., 2010), *Oryza sativa* (Morin et al., 2008), *Populus* spp. (Lu et al., 2005), and *Zea mays* (Wang et al., 2011). The second most abundant sequences were 23 nt in length, accounting for 13.81% of the sequences in the drought stress library, whereas sequences with a length of 20 nt were the second most abundant in the control and salt stress libraries, accounting for 19.50 and 18.16%, respectively.

### Identification of conserved miRNAs in the three libraries

Conserved miRNA families found in many species are responsive to many changes in the plant life cycle. To identify such conserved miRNAs in the three libraries, unique reads (minus reads mapping to snRNAs, snoRNAs, tRNAs, intron sequences, and coding sequences) from the libraries were subjected to a homolog search against miRBase21.

**Figure 2:**
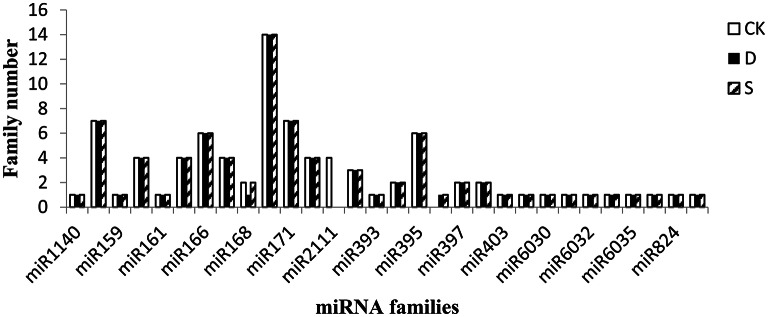
**Number of family members per miRNA family detected in the three libraries**. Candidate miRNA families were considered together and grouped by their miRBase numerical identifiers. CK, control; D, drought stress; S, salt stress.

In the CK, D, and S libraries, 85, 81, and 81 known miRNAs, respectively, were found, belonging to 29, 29, and 28 different miRNA families, respectively, with an average of 3 miRNA members per family among the three libraries (Table S4). The miRNA members of known families were highly divergent. Briefly, the miR169 family was the most abundant, with 14 members distinguished by internal nucleotide differences in the CK, S, and D small-RNA libraries, and 6–7 members of the miR156, miR166, miR171, and miR395 families were also found. Of the remaining miRNA families, 7 families had 2–4 members and 15 families had a single member in at least one library (Figure 2).

**Figure 3:**
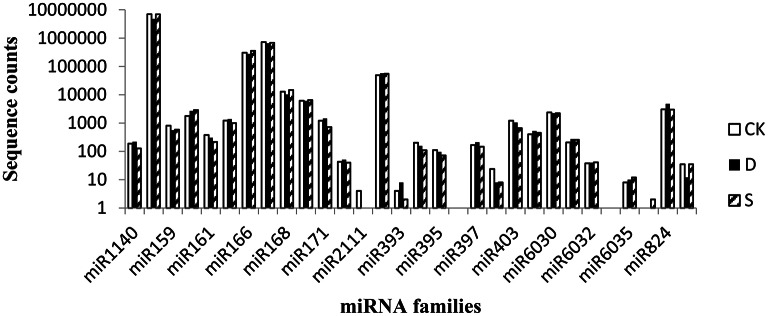
**Abundance of conserved miRNAs in the CK, D, and S libraries in rapeseed**. CK, control; D, drought stress; S, salt stress.

The relative abundance of miRNAs can be estimated by the number of reads revealed by high-throughput sequencing. Intriguingly, the rapeseed miRNA abundance exhibited substantial variation. For example, miR156, with 6,955,462, 4,784,853, and 6,920,959 redundancies in the CK, D, and S libraries, respectively, was the miRNA most frequently sequenced in the three libraries; in contrast, many miRNAs (e.g., miR6035, miR393, miR6034, miR2111, miR6036, and miR396) were sequenced <20 times (Figure 3, Table 2). Our sequence analysis indicated that the abundance of certain members of miRNA families can vary dramatically among the three libraries, suggesting functional variation within each family. As an example, the abundance of the miR168 family in the CK library varied from 1 read (miR168b) to 12,878 reads (miR168a) based on deep sequencing. Similar instances were found in other miRNA families, such as the miR156 (62,938–1,698,631 reads), miR167 (2785–240,853 reads), miR169 (8–2565 reads), and miR171 (6–592 reads) families. Such variation was similar in the other two libraries (Table S5), suggesting that distinct members in the same miRNA family have dramatically different levels of expression, likely due to tissue-specific, developmental stage-specific, or environment-specific expression.

**Table 2:** **The miRNA abundance of conserved miRNA families among control (CK), salt (S), and drought (D) treatments**.

**miR_name**	**Count**			**Fold change log**_**2**_ **Ratio normalized**			**Significance**		
	**CK**	**D**	**S**	**D/CK**	**S/CK**	**S/D**	**D/CK**	**S/CK**	**S/D**
bna-miR156f	1,698,631	1,178,297	1,692,709	−0.58	−0.11	0.48			
bna-miR156d	1,691,684	1,172,845	1,685,873	−0.58	−0.11	0.48			
bna-miR156a	1,690,785	1,172,163	1,685,001	−0.58	−0.11	0.48			
bna-miR156e	1,685,364	1,167,914	1,679,624	−0.59	−0.11	0.48			
bna-miR167a	240,853	217,836	228,063	−0.20	−0.18	0.02			
bna-miR167b	240,853	217,836	228,062	−0.20	−0.18	0.02			
bna-miR167c	240,687	217,691	227,973	−0.20	−0.18	0.02			
bna-miR156b	63,030	31,238	59,290	−1.07	−0.19	0.88	^*^		
bna-miR156c	63,030	31,237	59,289	−1.07	−0.19	0.88	^*^		
bna-miR156g	62,938	31,159	59,173	−1.07	−0.19	0.88	^*^		
bna-miR166e	58,893	53,340	68,334	−0.20	0.11	0.31			
bna-miR166b	58,893	53,337	68,334	−0.20	0.11	0.31			
bna-miR166c	58,893	53,337	68,333	−0.20	0.11	0.31			
bna-miR166a	58,877	53,342	68,310	−0.20	0.11	0.31			
bna-miR166d	58,786	53,247	68,200	−0.20	0.11	0.31			
bna-miR390b	16,224	19,115	18,376	0.18	0.08	−0.10			
bna-miR390a	16,222	19,111	18,371	0.18	0.08	−0.10			
bna-miR390c	16,222	19,111	18,371	0.18	0.08	−0.10			
bna-miR168a	12,878	10,166	14,762	−0.40	0.10	0.49			
bna-miR166f	11,544	10,656	13,676	−0.17	0.14	0.31			
bna-miR824	3047	4751	2977	0.58	−0.14	−0.72			
bna-miR167d	2785	2116	2489	−0.45	−0.26	0.19			
bna-miR169n	2565	3057	2933	0.20	0.09	−0.11			
bna-miR6030	2363	2238	2211	−0.13	−0.20	−0.06			
bna-miR169a	1310	1275	1439	−0.10	0.03	0.13			
bna-miR169b	1299	1269	1436	−0.09	0.04	0.13			
bna-miR403	1216	1048	661	−0.27	−0.98	−0.71			
bna-miR159	807	561	583	−0.58	−0.57	0.01			
bna-miR171f	592	673	344	0.13	−0.88	−1.01			^*^
bna-miR171g	592	673	344	0.13	−0.88	−1.01			^*^
bna-miR164a	493	546	403	0.09	−0.39	−0.48			
bna-miR160b	454	682	729	0.53	0.58	0.05			
bna-miR160d	454	682	729	0.53	0.58	0.05			
bna-miR160a	453	686	731	0.54	0.59	0.05			
bna-miR160c	426	613	683	0.47	0.58	0.11			
bna-miR6029	400	521	453	0.33	0.08	−0.25			
bna-miR161	380	303	216	−0.38	−0.92	−0.53			
bna-miR164b	245	278	199	0.13	−0.40	−0.53			
bna-miR164d	245	278	199	0.13	−0.40	−0.53			
bna-miR164c	243	277	199	0.13	−0.39	−0.52			
bna-miR6031	208	273	257	0.34	0.20	−0.13			
bna-miR1140	189	219	127	0.16	−0.68	−0.83			
bna-miR169g	148	68	106	−1.18	−0.58	0.59	^*^		
bna-miR169h	148	68	106	−1.18	−0.58	0.59	^*^		
bna-miR169k	148	68	106	−1.18	−0.58	0.59	^*^		
bna-miR169i	147	68	105	−1.17	−0.59	0.58	^*^		
bna-miR169j	147	68	105	−1.17	−0.59	0.58	^*^		
bna-miR169l	147	68	105	−1.17	−0.59	0.58	^*^		
bna-miR394a	101	78	55	−0.43	−0.98	−0.55			
bna-miR394b	100	78	55	−0.41	−0.96	−0.55			
bna-miR397a	84	105	73	0.27	−0.30	−0.57			
bna-miR397b	84	105	73	0.27	−0.30	−0.57			
bna-miR169m	40	31	23	−0.42	−0.90	−0.48			
bna-miR6032	38	40	41	0.02	0.01	−0.01			
bna-miR395a	35	31	21	−0.23	−0.84	−0.61			
bna-miR395b	35	31	21	−0.23	−0.84	−0.61			
bna-miR395c	35	31	21	−0.23	−0.84	−0.61			
bna-miR860	35	12	35	−1.60	−0.10	1.50	^*^		^*^
bna-miR172b	24	24	19	−0.06	−0.44	−0.38			
bna-miR399a	12	4	4	−1.64	−1.69	−0.05	^*^	^*^	
bna-miR399b	12	4	4	−1.64	−1.69	−0.05	^*^	^*^	
bna-miR172c	11	15	8	0.39	−0.56	−0.95			
bna-miR6035	8	10	12	0.27	0.48	0.22			
bna-miR169c	8	4	6	−1.06	−0.52	0.54	^*^		
bna-miR169d	8	4	6	−1.06	−0.52	0.54	^*^		
bna-miR169e	8	4	6	−1.06	−0.52	0.54	^*^		
bna-miR169f	8	4	6	−1.06	−0.52	0.54	^*^		
bna-miR171d	7	22	7	1.60	−0.10	−1.70	^*^		^*^
bna-miR171e	7	22	7	1.60	−0.10	−1.70	^*^		^*^
bna-miR171b	7	20	8	1.46	0.09	−1.37	^*^		^*^
bna-miR172a	7	8	11	0.14	0.55	0.41			
bna-miR171a	6	20	7	1.68	0.12	−1.56	^*^		^*^
bna-miR171c	6	20	7	1.68	0.12	−1.56	^*^		^*^
bna-miR393	4	8	2	0.94	−1.10	−2.05		^*^	^*^
bna-miR395d	2	1	3	−1.06	0.48	1.54	^*^		^*^
bna-miR395e	2	1	3	−1.06	0.48	1.54	^*^		^*^
bna-miR395f	2	1	3	−1.06	0.48	1.54	^*^		^*^
bna-miR172d	1	4	2	1.94	0.90	−1.05	^*^		^*^
bna-miR6036	1	1	2	−0.06	0.90	0.95			
bna-miR6034	1	1	0	−0.06	–	–			
bna-miR168b	1	0	1	–	−0.10	–			
bna-miR2111a-5p	1	0	0	–	–	–			
bna-miR2111b-3p	1	0	0	–	–	–			
bna-miR2111b-5p	1	0	0	–	–	–			
bna-miR2111d	1	0	0	–	–	–			
bna-miR396a	0	1	1	–	–	–			

Significant (^*^) if |log_2_ fold change| ≥ 1.0 and P ≤ 0.05; otherwise insignificant. CK, control; D, drought stress; S, salt stress.

Based on the criterion, |log_2_ fold change| ≥ 1.0 and *P* ≤ 0.05, the expression of miR166, miR167, and miR390, which were among the top 10 most expressed miRNAs, did not show significant differential expression. Indeed, only six miRNAs (miR156, miR169, miR171, miR395, miR399, and miR860), which were sequenced <50 times, were significantly differentially expressed between the drought stress and control treatments. Compared with salt stress, four miRNAs (miR171, miR393, miR395, and miR860) were markedly differentially expressed in the D library, and miR393 and miR399 were significantly differentially expressed in the CK library (Table 2). The miRNA family groups with similar expression patterns were then classified using Heatmap clustering. For instance, miR161, miR394, miR395, and miR403 were down-regulated by salt stress, whereas miR171, miR393, and miR824 were up-regulated by drought stress (Figure S1).

To explore the roles of these conserved miRNAs in evolution, further analyses focusing on broader comparisons against known conserved miRNAs in Viridiplantae, including Brassicaceae, Malvaceae, Rutaceae, Solanaceae, Salicaceae, Rhizophoraceae, and Lamiales, and even monocotyledons, Embryophyta, Coniferophyta, and Chlorophyta, were performed. Based on BLAST analysis, some miRNA families, such as miR156/157, miR159, miR160, miR165/166, miR171, and miR396, were found to be highly conserved (Table S6). For example, miR156/157 is highly expressed during the seedling stage in different species.

### Identification of novel miRNAs in rapeseed

**Figure 4:**
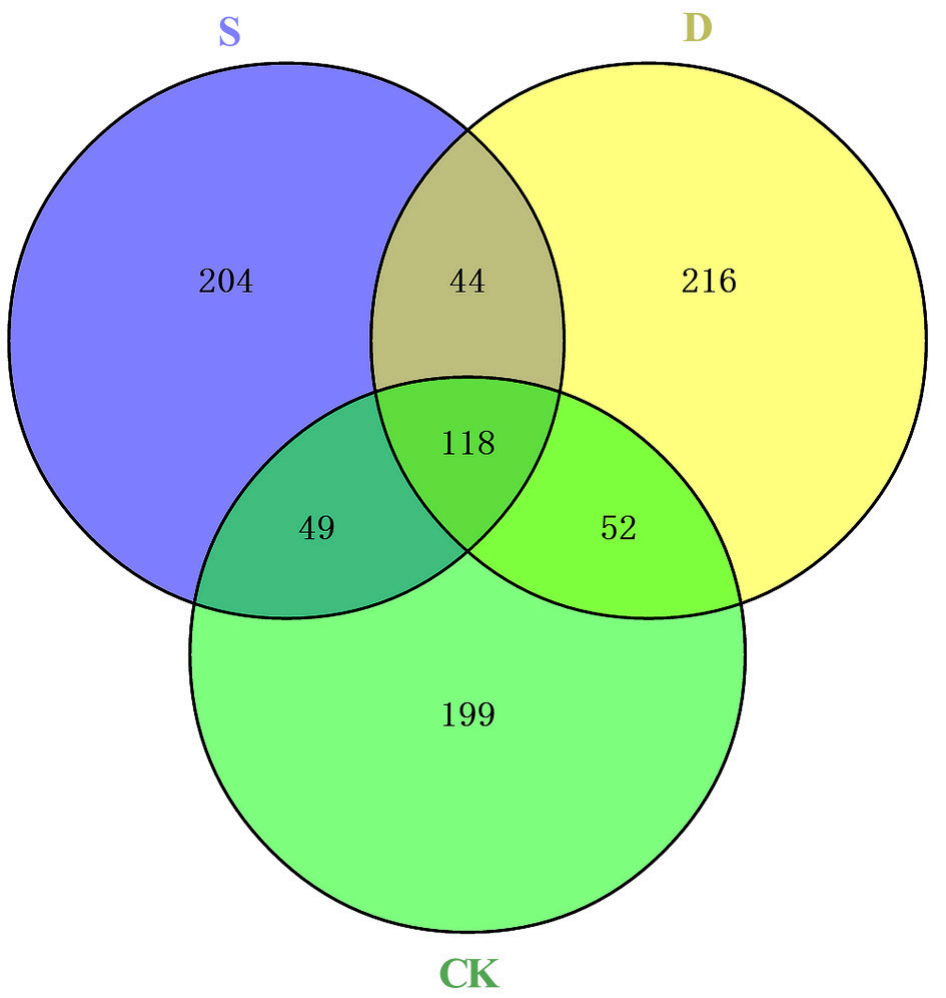
**Diagram of novel miRNAs identified in three libraries**. CK, control; D, drought stress; S, salt stress.

Each species possesses species-specific miRNAs (Zhao et al., 2010; Chi et al., 2011; Li et al., 2011; Schreiber et al., 2011; Song et al., 2011; Gao et al., 2012; Guo et al., 2012; Lertpanyasampatha et al., 2012; Lv et al., 2012), and the characteristic hairpin structures of miRNA precursors, the Dicer cleavage site, and the minimum free energy are important factors for predicting novel miRNAs. Many papers have reported the prediction of novel miRNAs using Mireap according to strict criteria (Li et al., 2013), and based on these criteria, 882 novel miRNAs were predicted from the three libraries (Table S7). The lengths of the novel miRNAs were 20–23 nt, with 23 nt being the most common length in the three libraries (Table S8). More than half of the novel predicted miRNAs begin with a 5′ uridine, and these miRNAs accounted for more than eighty percent of small RNAs 20 and 21 nt in length (Table S7) (Bonnet et al., 2004; Zhang et al., 2006). According to Mfold3.2, the average negative minimal folding free energy of these miRNA precursors is approximately −245 to −18 kcal mol^−*l*^, which was similar to miRNA precursors in other species (Wang et al., 2011). Compared with known miRNA families, the abundance of novel miRNAs was very low, and the majority of these miRNAs were present <100 times. Nonetheless, these miRNAs comprised 87.79% (367/418), 88.83% (382/430), and 88.92% (369/415) of the CK, D, and S libraries, respectively. The most abundant novel miRNA was novel_mir_34, which was sequenced 58,924, 70,723, and 60,320 times in the CK, D, and S libraries, respectively. Unlike conserved miRNAs, different types of novel miRNAs were expressed in the three independent libraries. Of the novel miRNAs identified, 199, 216, and 204 were specific to the CK, D, and S libraries, respectively, and only 118 were shared among the libraries. In the CK and D libraries, 170 novel miRNAs were shared, whereas 167 and 162 miRNAs were shared between the CK and S and D and S libraries, respectively (Figure 4).

**Figure 5:**
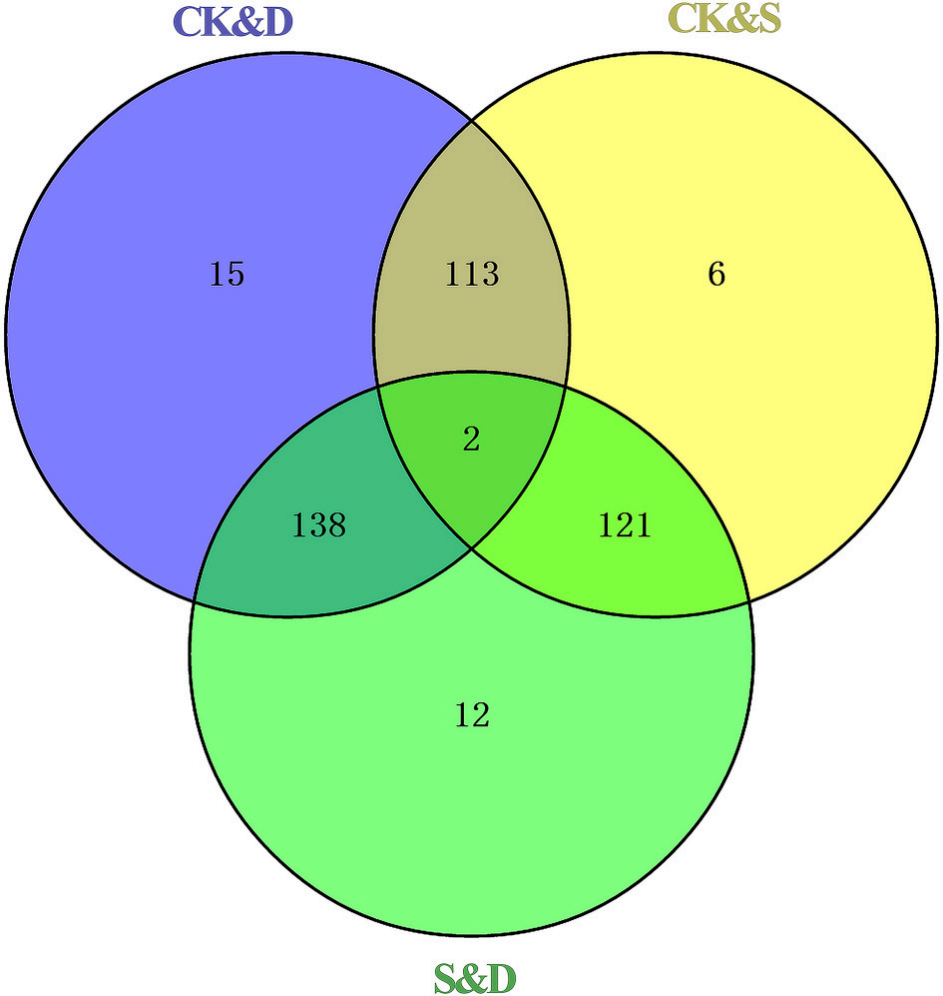
**Diagram of significantly different expression novel miRNAs between CK&D, CK&S, and S&D**. CK, control; D, drought stress; S, salt stress.

Using the criteria of |log_2_ fold change| ≥ 1.0 and *P* ≤ 0.05, 268, 242, and 273 miRNAs exhibited significantly different expression between the CK and D, CK and S, and S and D libraries, respectively. Two novel miRNAs—bna-miR25 and bna-miR385—were significantly up-regulated under drought stress and down-regulated under salt stress, respectively (Figure 5).

### Target prediction of rapeseed miRNAs

As the identification of mRNA targets is necessary for an understanding of the biological roles of *B. napus* miRNAs (Korbes et al., 2012), psRobot software was used to predict the targets of conserved and novel miRNAs. The cut-off threshold was set at 2.5, and 610 and 4133 putative targets were found for the conserved and 656 novel miRNA families, respectively (Tables S9, S10). However, no target genes were found for 226 of the novel miRNA families (Table S11). Various transcription factors, including SBP, MYB, ARF, NAM, NAC, CBF, TCP, NF-YA, and GRF, were among the majority of the conserved miRNA targets identified in different species, including *Arabidopsis* (Adai et al., 2005), grape (Carra et al., 2009), mustard (Xie et al., 2007), poplar (Lu et al., 2008), rice (Sunkar et al., 2005), soybean (Subramanian et al., 2008), and wheat (Jin et al., 2008). F-box protein, ATP sulfurylase, CCHC-type zinc finger protein, NAD(P)-binding protein, and Poly(ADP-ribose) polymerase, proteins involved in the regulation of metabolic processes, were also identified as conserved miRNA targets. In our datasets, miRNA156 showed the highest abundance, followed by miRNA167, miRNA166, and miRNA390, during the very early stage of seed germination under salt and drought stresses. miRNA156 is involved in floral development and phase change through down-regulation of the SQUAMOSA promoter binding protein-like (SPL) family of transcription factor genes, which are up-regulated in the juvenile phase and down-regulated in the adult phase (Wu et al., 2009; Zhang et al., 2009; Yang et al., 2011). Many studies have emphasized that the transcription factor HD-ZIP participates in plant leaf morphogenesis, and *ATHB15*, a member of the HD-ZIP family targeted by miRNA166, may play a role in plant vascular development because it is predominantly expressed in vascular tissues (Ohashi-Ito and Fukuda, 2003). The miRNA156 and miRNA166 families are very important in seed germination not only due to their high abundance but also because of the large size of those miRNA families compared to other miRNA families. Studies have demonstrated cross-talk regulation between ABA and auxin during seed germination (Weitbrecht et al., 2011), and miRNA167 and miRNA160 have been shown to be involved in inhibiting auxin response factors ARF6 and ARF8 (Wu et al., 2006) and ARF10, ARF16, and ARF17 (Mallory et al., 2005; Wang et al., 2005), respectively. Additionally, miRNA159 has been shown to be involved in the regulation of seed dormancy and germination by inhibiting *MYB33* and *MYB101*, two positive regulators of ABA response during germination.

To gain further insight into the functions of novel miRNAs in rapeseed during seed germination under salt and drought stresses, putative targets of the 656 newly identified miRNAs were predicted, revealing various functions, including transcription, metabolism, transport, kinases, oxidative reduction and isomerase and helicase activities. According to miRNA regulatory network analysis using Cytoscape (http://www.cytoscape.org/), 271 miRNA families and 20 target gene families in rapeseed were confirmed to be core miRNAs and genes involved in response to salt and drought stresses. Direct stress-responsive gene families, such as disease resistance protein (DIRP), drought-responsive family protein (DRRP), early responsive to dehydration stress protein (ERD), stress-responsive alpha-beta barrel domain protein (SRAP), and salt tolerance homolog2 (STH2), were also predicted in our analysis. Overall, miRNA regulatory mechanisms responsive to salt and drought stresses are highly sophisticated in rapeseed, and these miRNAs and their targets related to stress response add to our knowledge of stress resistance and the stress tolerance mechanism in this plant.

### Expression profiles of miRNAs and their targets in response to salt and drought stresses

**Figure 6:**
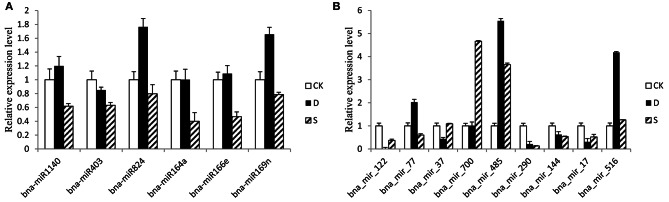
**Quantitative RT-PCR validation of mature miRNAs**. The expression profiles of mature miRNAs were consistent with the results obtained by small-RNA deep sequencing. The expression values presented are the means of three technical replicates. U6 was used for each sample as an endogenous control. **(A,B)** Represent the expression pattern of mature conserved and novel miRNAs, respectively.

**Figure 7:**
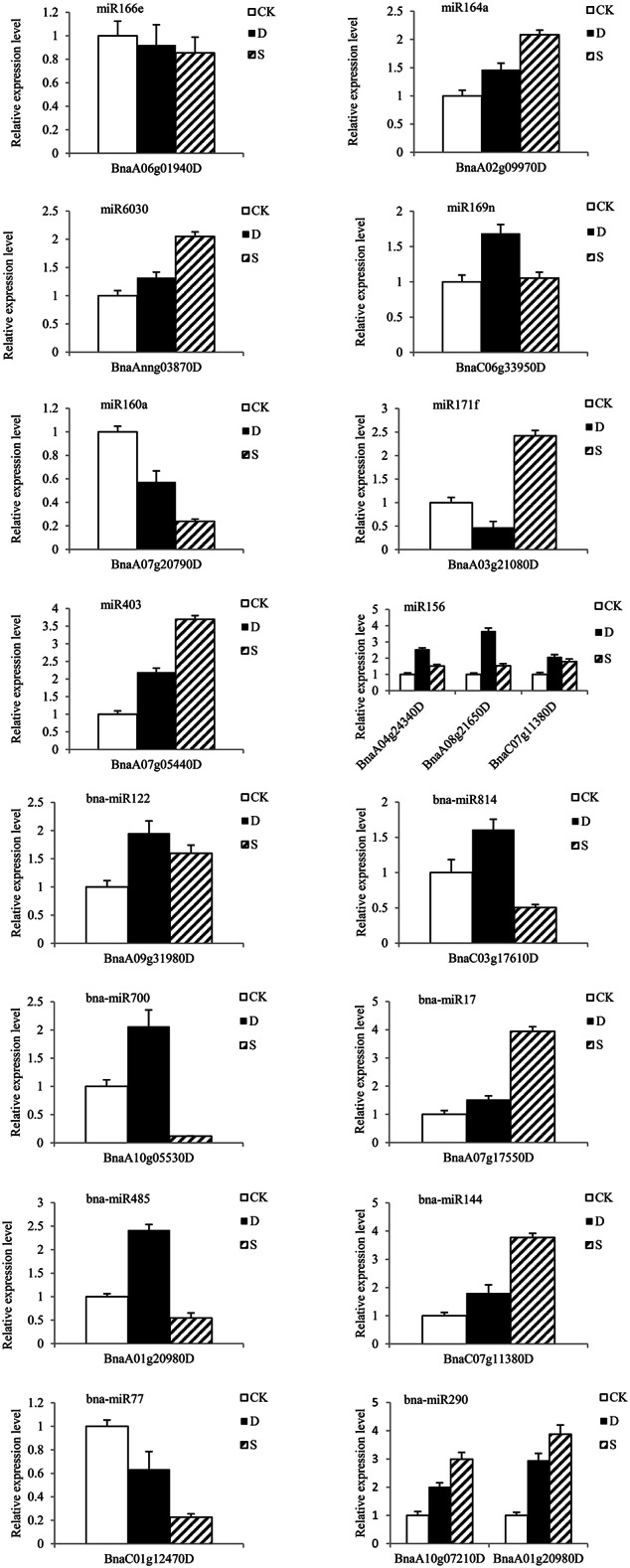
**Quantitative RT-PCR analyses of relative expression levels of various predicted target genes**. The rapeseed housekeeping gene actin7 was used as the internal control. The values presented are the means of three technical replicates.

To confirm the expression patterns of miRNAs in response to salt and drought stresses, quantitative RT-PCR (qRT-PCR) was performed for 6 conserved miRNAs (miR1140, miR403, miR824, miR164a, miR166e, and miR169n) and 9 novel ones (bna-miR17, bna-miR37, bna-miR77, bna-miR122, bna-miR144, bna-miR290, bna-miR485, bna-miR516, and bna-miR700). As expected, the qRT-PCR results were consistent with the expression profiles obtained by RNA-seq (Figure 6). qRT-PCR was also performed on target genes of 17 randomly selected miRNAs, of which 8 are from known miRNA families (miR156b, miR403, miR160a, miR164a, miR166e miR171f, miR6030, and miR169n) and 9 from novel miRNA families (bna-miR17, bna-miR37, bna-miR77, bna-miR122, bna-miR144, bna-miR290, bna-miR485, bna-miR700, and bna-miR814). However, as illustrated in Figure 7, the expression profiles of 19 target genes showed an inverse relationship with the expression profiles of their corresponding miRNAs, suggesting that the miRNAs indeed target those genes.

## Discussion

Seed germination is a critical step in plant growth and development. However, abiotic stresses such as drought and salt are major factors affecting the seed germination rate. Thus, understanding this process as a whole—from early seed germination to the establishment of the seedling—can help us to engineer and select more robust crop species and increase crop yield and quality. In this study, three sRNA libraries from imbibed seed embryos under control, salt, and drought treatments were constructed, and miRNAs were sequenced using high-throughput sequencing.

### Many miRNAs participate in rapeseed germination

High-throughput sequencing has been applied to study miRNAs at the whole-genome level in many plant species. However, the means by which miRNA families function in plant development, stress responses, and other processes in *B. napus*, one of the most important oil crops in the world, are not very clear. In our study, the three libraries contained 35,322,868 clean reads and 14,084,059 unique small-RNA reads. Two of the three libraries shared from 65.27 to 67.88% of the total clean reads; however, they shared only 14.34–14.68% of the unique small-RNA reads, a phenomenon also observed in maize (Li et al., 2013). These findings suggest that the shared reads have high expression levels and the library-specific small RNAs low expression levels. The low expression levels of these specific unique small RNAs may indicate that they function in specific and unique regulation pathways.

Successful seed germination and the establishment of a normal seedling are essential. Although the evidence is fragmentary, there are several sources that emphasize miRNAs as a very important component of seed germination (Li et al., 2013; Zhang et al., 2013). For instance, Weitbrecht et al. (2011) highlighted that ABA and gibberellin acid (GA) play vital roles in seed germination. Briefly, ABA levels generally decrease during imbibition, whereas GA levels increase during the transition to germination. In *Arabidopsis*, two positive ABA regulators, *MYB33* and *MYB101*, which are negatively regulated by miR159, play key roles in seed germination. In the present study, *BnaA04g18810D* and *BnaC04g43020D*, homologs of *AtMYB101*, were predicted to be target genes of miR159. In the absence of miR159, *MYB33*, and *MYB101* up-regulated genes are deregulated during seed germination (Alonso-Peral et al., 2010); thus, *MYB-like* genes participate in GA-induced pathways via miR159-mediated regulation during seed germination. AUXIN RESPONSIVE FACTORs (ARFs), which are pivotal for translating auxin signals into transcriptional responses, comprise a class of targets for the miRNA160/167 families. Indeed, studies have shown that the negative regulation of ARF10 by miR160 plays an important role in seed germination (Liu et al., 2007), and miR167 regulates ARF6 and ARF8 in adventitious rooting in *Arabidopsis* (Gutierrez et al., 2009) and in cultured rice cells (Yang et al., 2006). The crosstalk between ABA and auxin in imbibed mature seeds has been discussed by Liu et al. (2007), and studies have indicated that ABA sensitivity would decrease in mature seeds via the down-regulation of auxin signal transduction. Regardless, the mechanisms of ABA-auxin crosstalk during seed germination remain enigmatic. Laccases, which are involved in the lignification and thickening of the cell wall during secondary cell growth (Constabel et al., 2000), were found to be down-regulated by miR397, suggesting that miR397 plays a vital role in decreasing the thickness of the cell wall during the transition from a dormant embryo to a germinated embryo by causing laccase mRNA cleavage. Other miRNA families have been reported to be involved in seed germination in other species, such as miR166 (*HD-ZIP*), miR164 (*NAC1*), and miR396 (*GRF*) (Tahir et al., 2006; Li et al., 2013; Zhang et al., 2013). These findings indicate the existence of complex mechanisms of gene regulation involving miRNAs during seed germination.

### Potential roles of miRNAs in seed germination response to salt and drought stresses

Many conserved and novel miRNAs are aberrantly expressed in drought stress in different crops, such as cowpea (Barrera-Figueroa et al., 2011), tobacco (Frazier et al., 2011), wheat (Kantar et al., 2011), soybean (Kulcheski et al., 2011), and bean (Arenas-Huertero et al., 2009). miR169, one of the most important miRNAs involved in drought stress, was down-regulated in our study. Similarly, the expression of miR169a and miR169c in *Arabidopsis* decreased under drought stress, and the miR169-target gene *NFYA5*, a member of the *Arabidopsis* family of CCAAT-box nuclear transcription factors functioning in drought resistance, was strongly induced (Li et al., 2008). However, miR169 was found to be up-regulated in rice and tomato under drought conditions (Zhao et al., 2007; Zhang et al., 2011). The three nuclear factor Y subunit genes (SlNF-YA1/2/3) targeted by miR169 were also down-regulated. Moreover, overexpressing miR169c in tomato plants resulted in better tolerance to drought. In *Arabidopsis*, miR157, miR167, miR168, miR171, miR408, miR393, and miR396 were reported to be up-regulated in response to drought stress (Liu et al., 2008). In our study, miR156b/c/g, miR171a/d/e, and miR172d were significantly up-regulated, whereas miR860, miR399a/b, and miR395d/e/f were down-regulated.

Although plants invoke similar cellular and metabolic responses to salt and drought stresses (Munns, 2005), the genes and pathways involved vary (Bartels and Sunkar, 2005; Golldack et al., 2011). In our study, miR393 and miR399, both are involved in phosphate homeostasis, were significantly down-regulated. However, miR399 was up-regulated under low phosphate in *Arabidopsis* (Fujii et al., 2005) and rice (Wang et al., 2009). When plants suffer from phosphate starvation, mature miR399 activated by the PHR1 transcription factor cleaves target PHO2 mRNA (Pant et al., 2008). In addition, mRNAs of TIR1/AFB2 auxin receptors (TAARs), regulators of auxin signaling homeostasis, are targeted and cleaved by up-regulated miR393 during salt and drought stresses in *O. sativa, A. thaliana, M. truncatula*, and *Phaseolus vulgaris* (Dharmasiri et al., 2005; Chen et al., 2011; Si-Ammour et al., 2011; Windels and Vazquez, 2011). miR393 also functions in the root development response to nitrate treatment by cleaving TAAR transcripts, which are positive regulators of auxin signaling (Vidal et al., 2010). It is easy to understand the crucial rule of miR393 in response to different stresses because auxin is involved in almost every aspect of plant life (Windels and Vazquez, 2011). These miRNAs may increase drought or salt tolerance through unknown mechanisms associated with crosstalk regulation in plants, and future studies in rapeseed involving miRNA over-expression for functional annotation are warranted.

## Author contributions

LL conceived and designed the experiments. HJ and JW performed the experiments. HJ, JW, and TW analyzed the data. LW, JL, and LL contributed reagents/materials/analysis tools. HJ wrote the paper.

## Funding

This study was supported by National Natural Science Foundation of China (31371655), 973 Program (2015CB150201), and Chongqing graduate student research innovation project (CYB2015063).

### Conflict of interest statement

The authors declare that the research was conducted in the absence of any commercial or financial relationships that could be construed as a potential conflict of interest. The reviewer AVCR and handling Editor declared their shared affiliation, and the handling Editor states that the process nevertheless met the standards of a fair and objective review.

## Supplementary material

Table S1 **The primers used for mature miRNAs qRT-PCR**.

Table S2 **The primers used for predicted miRNAs targets qRT-PCR**.

Table S3 **Raw data analysis for the three libraries**.

Table S4 **The number of conserved miRNA family members**.

Table S5 **The miRNA abundance of conserved miRNA families among the control (CK), salt (S), and drought (D) treatments**.

Table S6 **Conserved miRNAs from *B. napus* and their homologs in selected other species**. +, detected in this species; −, not detected in this species.

Table S7 **The miRNA abundance of novel miRNA families among the control (CK), salt (S), and drought (D) treatments**.

Table S8 **Size distribution of novel miRNAs in the rapeseed CK, D, and S libraries**.

Table S9 **Predicted targets of conserved miRNAs in the CK, D, and S libraries**.

Table S10 **Predicted targets of novel miRNAs in the CK, D, and S libraries**.

Table S11 **Novel miRNAs with no targets predicted in the CK, D, and S libraries**.

Figure S1 **Heat map of conserved miRNA read abundance in the *B. napus* control, salt, and drought libraries**.
