# Supplementary material for: Identification of Rapeseed MicroRNAs Involved in Early Stage Seed Germination under Salt and Drought Stresses
Source: Front Plant Sci. 2016 May 13;7:658. doi: 10.3389/fpls.2016.00658 (PMC4865509; doi:10.3389/fpls.2016.00658)
Supplement: Table S1 — The primers used for mature miRNAs qRT-PCR. [file Table1.DOCX]

**Table S1: Primers used for mature miRNAs qRT-PCR**

| Table S1 Primers used for mature miRNAs qRT-PCR | |  |
| --- | --- | --- |
| miRNA name | Primer | Sequence (5'-3') |
| bna-miR1140 | Forward primer | ACAGCCTAAACCAATCGGAGC |
| bna-miR156b | Forward primer | TTGACAGAAGATAGAGAGCAC |
| bna-miR160a | Forward primer | TGCCTGGCTCCCTGTATGCCA |
| bna-miR164a | Forward primer | AGAAGCAGGGCACGTGCA |
| bna-miR166e | Forward primer | GACCAGGCTTCATTCCCCA |
| bna-miR167b | Forward primer | TGAAGCTGCCAGCATGATCTAA |
| bna-miR168a | Forward primer | CGCTTGGTGCAGGTCGG |
| bna-miR169n | Forward primer | CAGCCGAGGATGACTTGCC |
| bna-miR171f | Forward primer | GAGCCGCGCCAATATCAA |
| bna-miR403 | Forward primer | CCTTAGATTCACGCACAAACTCG |
| bna-miR6030 | Forward primer | TCCACCCATACCATACAGACCC |
| bna-miR824 | Forward primer | CGTAGACCATTTGTGAGAAGGGA |
| novel_mir_122 | Forward primer | TGGTGATTGAGCCGCGTC |
| novel_mir_485 | Forward primer | CGCTGGAGTAGCTCAGTTGGTT |
| novel_mir_290 | Forward primer | CGCTGGAGTAGCTCAGTTGGTA |
| novel_mir_303 | Forward primer | TGTTGTAGAATTTTGGGAAGGGC |
| novel_mir_144 | Forward primer | CGTGCCTTGACAGAAGAGAGC |
| novel_mir_17 | Forward primer | CGTGGCGGTTGACAGAAGAA |
| novel_mir_516 | Forward primer | TCCACCAATGAAAGGTATGATTCC |
| novel_mir_337 | Forward primer | CGTCGGCTGACAGAAGAGAGG |
| novel_mir_77 | Forward primer | CGCACGGCAAGTTGTCCTTC |
| novel_mir_37 | Forward primer | TGCAGGAGAGATAGCGCCA |
| novel_mir_814 | Forward primer | CGTCCGTCGTAGTCTAGCTGGTT |
| novel_mir_700 | Forward primer | CGCTCGGCAAGTTGTCCTG |
| U6 | Forward primer | TTGGAACGATACAGAGAAGATTAGCA |

Reverse primer was obtained from the miRcute miRNA qPCR detection kit (FP401, Tiangen, Beijing, China).
