# Supplementary material for: Identification of Rapeseed MicroRNAs Involved in Early Stage Seed Germination under Salt and Drought Stresses
Source: Front Plant Sci. 2016 May 13;7:658. doi: 10.3389/fpls.2016.00658 (PMC4865509; doi:10.3389/fpls.2016.00658)
Supplement: Table S2 — The primers used for predicted miRNAs targets qRT-PCR. [file Table2.DOCX]

**Table S2**: Primers used for predicted miRNAs targets qRT-PCR

| miRNA name | Target | Primer (from 5' to 3') | |
| --- | --- | --- | --- |
|  |  | F | R |
| bna-miR156b | BnaA04g24340D | ATAGCACCTTCCCTTTACG | CTGGCTGATGTGGATTTG |
| bna-miR156b | BnaA08g21650D | TCCAGGTGAAACCATCCA | CTTCCGAGCAACAACAGA |
| bna-miR156b | BnaC07g11380D | CCGTGGAGATTCTTGCTG | GATGATTTGGATTTCTTTCG |
| bna-miR166e | BnaA06g01940D | CCGCTCTACGAGTCACCA | CCTATCACCGACCATCCT |
| bna-miR160a | BnaA07g20790D | CGAGTGGGTTCTTGAGTG | TGCCAGCAGGAAATGTAG |
| bna-miR171f | BnaA03g21080D | TTTGTTCAGATAGAGGGTG | TTCAATCCAACGGTGTC |
| bna-miR169n | BnaC06g33950D | CTGCGACTCATACTAAACC | TACCAGGAACCATACCC |
| bna-miR164a | BnaA02g09970D | TCATAACCACCGCAATAA | GACGACCCATAGTAAGGAA |
| bna-miR403 | BnaA07g05440D | CGGCACTGACTCACCAAAC | TCGTACCGTCCATCACCAC |
| bna-miR6030 | BnaAnng03870D | GCCTATTCAACCCACA | ACCATACAAACCCAAA |
| novel_mir_122 | BnaA09g31980D | GTCTATCCCAGAAACTACTAAA | ATGCTATTCGAGCTTGTC |
| novel_mir_485 | BnaA01g20980D | CAAAGACTCGGCAAACAC | AGCAAGACCCACCTCAAC |
| novel_mir_290 | BnaA10g07210D | GGGACCGTGATGAAAGTA | AAGTATGCGTATCTGAGTGG |
| novel_mir_144 | BnaC07g11380D | CCGTGGAGATTCTTGCTG | GATGATTTGGATTTCTTTCG |
| novel_mir_17 | BnaA07g17550D | AGCAGGTTTCACTGTCTC | TCACTTCGTGGATGGATA |
| novel_mir_290 | BnaA01g20980D | CAAAGACTCGGCAAACAC | AGCAAGACCCACCTCAAC |
| novel_mir_77 | BnaC01g12470D | AGTGGTGGGTCTAAGGTCA | CATTGTAACAGCCAAGTGC |
| novel_mir_814 | BnaC03g17610D | GGACCAAGTTGCTGTGAGA | GCCAAGAAACAAGAGGGAT |
| novel_mir_700 | BnaA10g05530D | AAGAAACCGAACCCAAACA | CTTCACCCGACAATGCTAC |
|  | Actin | TGGGTTTGCTGGTGACGAT | TGCCTAGGACGACCAACAATACT |
