# Supplementary material for: Identification of Rapeseed MicroRNAs Involved in Early Stage Seed Germination under Salt and Drought Stresses
Source: Front Plant Sci. 2016 May 13;7:658. doi: 10.3389/fpls.2016.00658 (PMC4865509; doi:10.3389/fpls.2016.00658)
Supplement: Table S3 — Raw data analysis for the three libraries. [file Table3.DOCX]

**Table S3:** Raw data analysis in three libraries

| type | CK | | D | | S | |
| --- | --- | --- | --- | --- | --- | --- |
|  | count | % | count | % | count | % |
| total_reads | 11528557 |  | 12080081 |  | 12315608 |  |
| high_quality | 11468864 | 100 | 12016921 | 100 | 12251531 | 100 |
| 3'adapter_null | 18965 | 0.17 | 44398 | 0.37 | 16385 | 0.13 |
| insert_null | 3190 | 0.03 | 4675 | 0.04 | 4873 | 0.04 |
| 5'adapter_contaminants | 59152 | 0.52 | 22774 | 0.19 | 47602 | 0.39 |
| smaller_than_18nt | 67943 | 0.59 | 26319 | 0.22 | 92999 | 0.76 |
| polyA | 1699 | 0.01 | 1735 | 0.01 | 1739 | 0.01 |
| clean_reads | 11317915 | 98.68 | 11917020 | 99.17 | 12087933 | 98.66 |
