# Supplementary material for: Identification of Rapeseed MicroRNAs Involved in Early Stage Seed Germination under Salt and Drought Stresses
Source: Front Plant Sci. 2016 May 13;7:658. doi: 10.3389/fpls.2016.00658 (PMC4865509; doi:10.3389/fpls.2016.00658)
Supplement: Table S8 — Size distribution of novel miRNAs in the rapeseed CK, D, and S libraries. [file Table8.DOCX]

Table S8. Size distribution of novel miRNAs in CK, D and S libraries from oilseed
