# Supplementary figures and images for: Identification of Rapeseed MicroRNAs Involved in Early Stage Seed Germination under Salt and Drought Stresses
Source: Front Plant Sci. 2016 May 13;7:658. doi: 10.3389/fpls.2016.00658 (PMC4865509; doi:10.3389/fpls.2016.00658)

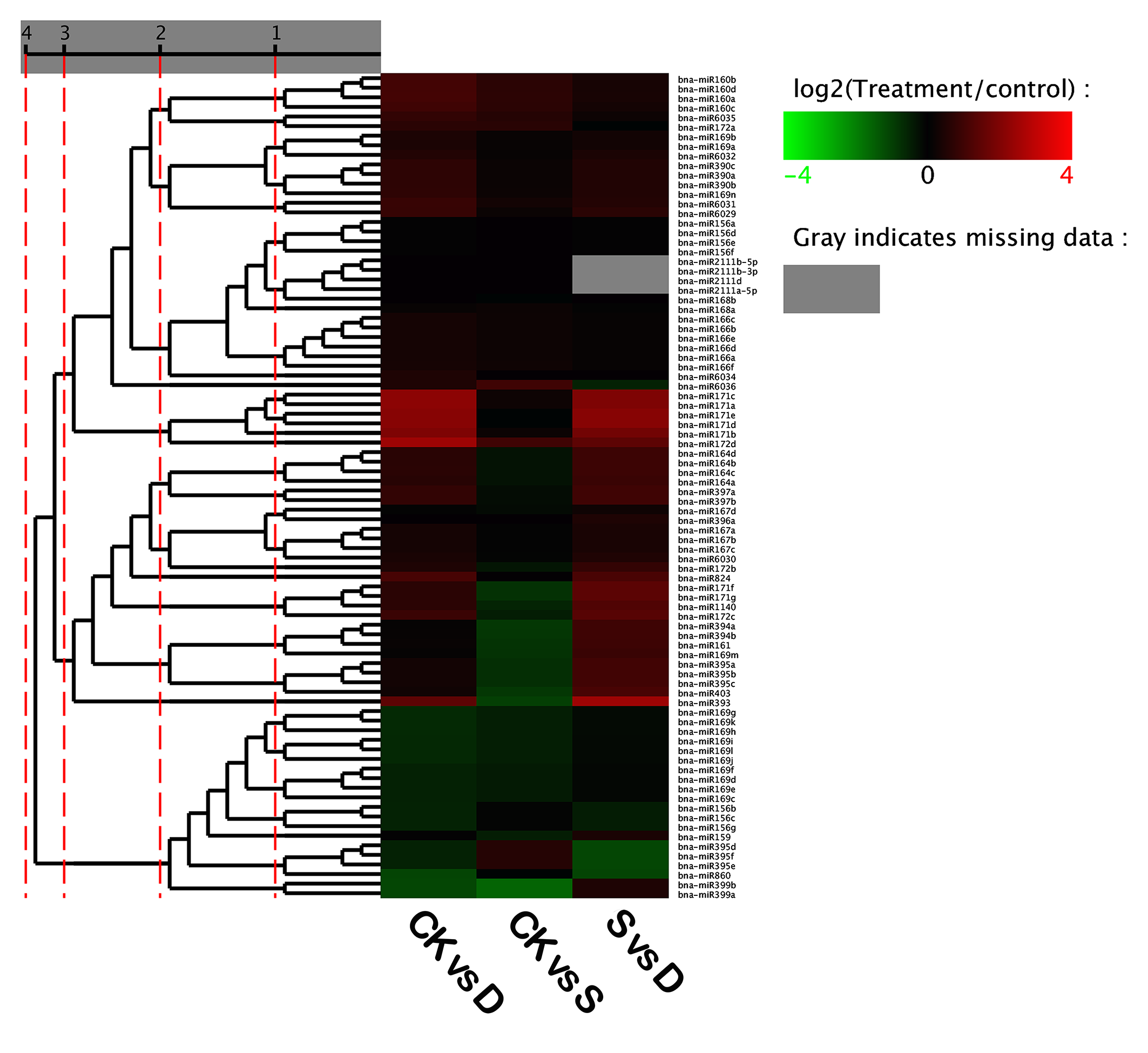

Supplement: Figure S1 — Heat map of conserved miRNA read abundance in the B. napus control, salt, and drought libraries. [file Image1.TIF]
